# Prevalence of Sleep Disorders Among the General Population of the Jazan Region of Southwest Saudi Arabia

**DOI:** 10.7759/cureus.46218

**Published:** 2023-09-29

**Authors:** Abdulrahman Hakami, Raghad A Hakami, Maryam A Al-Amer, Laila M Sharahili, Alhanouf H Zuqayl, Thanaa K Hakami, Ibrahim M Dighriri

**Affiliations:** 1 Department of Medicine, Faculty of Medicine, Jazan University, Jazan, SAU; 2 Faculty of Medicine, Jazan University, Jazan, SAU; 3 Department of Pharmacy, King Abdulaziz Specialist Hospital, Taif, SAU

**Keywords:** jazan, insomnia, osa, sleep apnea, sleep disorders

## Abstract

Background: Sleep disorders impose a substantial burden on the global population, leading to an array of health complications. Understanding their prevalence and associated risk factors is crucial to formulating effective interventions.

Objective: This study aimed to determine the prevalence and associated risk factors of sleep disorders among residents of the Jazan region of Saudi Arabia.

Methods: This cross-sectional study conducted an online survey from December 2022 to March 2023. The sample comprised 670 respondents aged 18 years and older residing in Jazan. Demographic data, lifestyle habits, sleep patterns, and sleep disorder symptoms were assessed.

Results: The participants were predominantly women (62.2%), with an average age of 30.99 years and a normal body mass index. The analyses revealed that 28.8% of the respondents reported experiencing sleep disorders, and 13.4% particularly had obstructive sleep apnea. Nearly half of the participants reported having primary insomnia, excessive daytime sleepiness, and restless leg syndrome. Significant associations were found between sleep disorders and older age (p = 0.012), obesity (p = 0.043), short or thin neck (p = 0.034), smoking (p = 0.003), caffeine use (p = 0.001), existing health conditions (p = 0.001), medication use (p = 0.013), lack of daytime naps (p = 0.043), and frequent nighttime awakenings to urinate (p = 0.001). The most common self-reported reasons for nightly awakenings were urination, anxiety or stress, discomfort or noise, and health conditions.

Conclusions: The findings suggest a high prevalence of sleep disorders among adults in Jazan, Saudi Arabia. Various demographic, lifestyle, and health-related factors are linked to these disorders. Therefore, targeted sleep health education and interventions could be instrumental in tackling this significant public health issue.

## Introduction

Sleep disorders are prevalent worldwide, affecting many people throughout their lives [1]. Estimates suggest that 50-70 million Americans chronically experience sleep and wakefulness disorders [2]. Sleep disorders are linked to adverse health, safety, economic, and quality of life consequences [3]. Insufficient sleep is associated with diabetes, heart disease, obesity, and depression [4].

Obstructive sleep apnea (OSA) is a common sleep disorder that affects 4-5% of middle-aged people [5]. It is caused by partial or complete upper airway closure during sleep, resulting in breathing cessations (apneas) or shallow breathing (hypopneas) [6]. Repeated apneas and hypopneas during sleep lead to intermittent hypoxia, hypercapnia, sleep disruption, and increased sympathetic activity [7,8].

Saudi Arabia likely has a high prevalence of sleep disorders, including OSA, owing to the increasing prevalence of obesity and diabetes [9,10]. Some Saudi studies have evaluated sleep disorder and OSA prevalence in Riyadh (21%), and the eastern province (26%) [11,12]. A study found that 52.4% of primary care patients are at high risk for OSA [13] in Jazan, southwest Saudi Arabia, which has a population of around 1.5 million people and a hot and tropical climate [14]. Due to its high prevalence of obesity and diabetes (over 30%) [15,16], Jazan, one of the regions of Saudi Arabia, is likely to have a high prevalence of sleep disorders, including OSA. However, data on sleep disorders in Jazan are currently lacking [17,18]. Therefore, this study aimed to determine sleep disorders' prevalence and risk factors in Jazan.

## Materials and methods

Study design

This quantitative descriptive cross-sectional study was conducted using a self-administered questionnaire distributed from December 2022 to March 2023.

Study setting and participants

This study was performed in the Jazan region of Saudi Arabia. The study population included all residents of the Jazan region who fulfilled the inclusion criteria. Participants who were more than 18 years old and living in the Jazan region were included. Conversely, participants who were less than 18 years old, who refused to participate, and who were living outside the Jazan region were excluded.

Sampling technique and sample size

We deployed a stratified random sampling technique to recruit the participants. To determine the sample size for this study, we utilized the Raosoft sample size calculator (Raosoft Inc., Seattle, WA, USA; http://www.raosoft.com). The tool indicated that a sample of 385 participants from the pool of 1,637,361 Jazan residents was necessary to secure a 95% confidence interval with a 5% margin of error.

Data collection

Data were collected across various parts of the Jazan region through an online self-administered questionnaire. The questionnaire was divided into two parts: The first section gathered information on sociodemographic factors (i.e., age, sex, and occupation), health-related data, and behavioral details. The second section collected information on sleep symptoms, situational sleepiness, sleep patterns, reasons for nighttime awakenings, and prevalence of sleep disorders. 

Pilot study

We initiated the study process by testing the data collection tool on a small sample of 15 individuals who were not part of the main study. This preliminary testing helped us assess the clarity and comprehensibility of the questionnaire and the time needed for completion and allowed us to rectify any errors. To ensure reliability, we relied on Cronbach’s alpha test, which was 0.9. Based on the feedback and test results, we made any necessary adjustments to the data collection tool to enhance its usability and effectiveness.

Data analysis

Upon completion of the data collection via the online questionnaire, the data were systematically coded and entered into the Statistical Package for the Social Sciences version 23.0 (IBM Corp., Armonk, NY) for analysis. We set a p-value threshold of <0.05 to test for significance. Furthermore, we used frequencies, percentages, standard deviations, and other means to represent and illustrate the data, ensuring a comprehensive understanding of the findings. The chi-square test was used to analyze the associations between the sociodemographic and lifestyle factors and sleep disorder prevalence. Figures and tables were created to visualize the distributions.

Ethical consideration

This study received approval from the Research Ethics Committee (REC) at Jazan University under reference number REC-44/05/408. As part of ethics compliance, we incorporated a consent question at the beginning of the questionnaire. When participants declined to provide consent, the questionnaire link automatically closed. However, when they agreed, they proceeded to answer the questionnaire, our primary data collection instrument. It was explained to all participants that they had the full right to withdraw at any study stage without any repercussions or loss of benefits. We took meticulous measures to preserve the personal information of the participants, ensuring absolute confidentiality throughout the study.

## Results

A total of 670 respondents participated in the study. Most were women (n = 417, 62.2%) and had a normal or long neck size (n = 588, 87.8%). The mean age of the respondents was 30.99 years, with a standard deviation of 11.66 years. The average height was 162.95 centimeters, and the average weight was 64.19 kilograms. Regarding occupation, the largest group comprised students (n = 262, 39.1%), followed by others or retired individuals (n = 224, 33.4%). Overweight and obesity were common (n = 184, 27.5%, and n = 112, 16.7%, respectively) based on the body mass index (BMI). Most respondents were non-smokers (n = 594, 88.7%) and did not regularly take medications (n = 521, 77.8%). High blood pressure was noted in the sample's 9.9% (n = 66) (Table 1).

**Table 1:** Sociodemographic characteristics of the respondents Abbreviation: SD: Standard deviation. N: Number. (%): Percentage.

Sociodemographic characteristics		n	%
Sex	Male	253	37.8
	Female	417	62.2
Neck circumference	Long or normal	588	87.8
	Short or thin	81	12.1
	Middle size	1	0.1
Occupation	Government sector employee	148	22.1
	Private sector employee	36	5.4
	Student	262	39.1
	Others or retired	224	33.4
Body mass index	Underweight	93	13.9
	Normal weight	281	41.9
	Overweight	184	27.5
	Obesity	112	16.7
Do you smoke?	Yes	76	11.3
	No	594	88.7
Do you take medicines?	Yes	149	22.2
	No	521	77.8
Do you have a high blood pressure?	Yes	66	9.9
	No	604	90.1
Age by year; mean ± SD		30.99 ± 11.66	
Height by centimeter; mean ± SD		162.95 ± 57.73	
Weight by kilogram; mean ± SD		64.19 ± 18.66	

**Figure 1:**
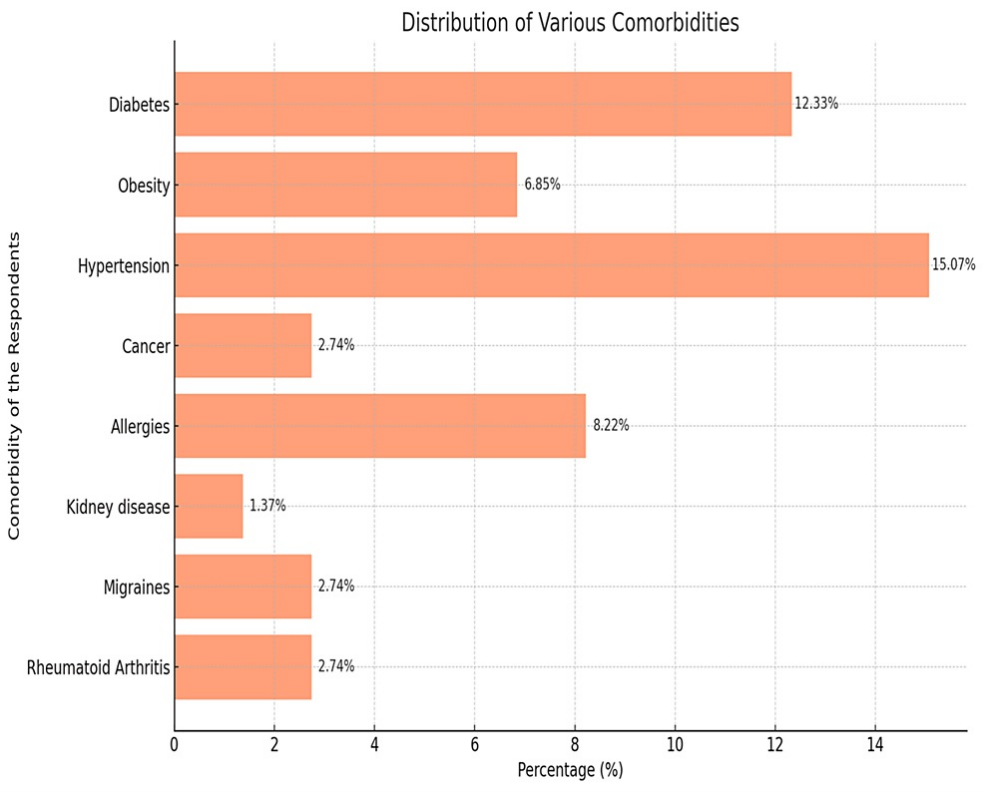
Comorbidity of the respondents

As shown in Figure 1, n = 83, 12.33% of the respondents indicated having diabetes; n = 101, 15.07% indicated having hypertension; and n = 46, 6.85% indicated having obesity. Other common comorbid conditions included allergies at n = 55, 8.22%, cancer at n = 18, 2.74%, and migraine at n = 18, 2.74%. Rheumatoid arthritis and kidney disease were reported by n = 18, 2.74%, and n = 9, 1.37%, respectively.

**Figure 2:**
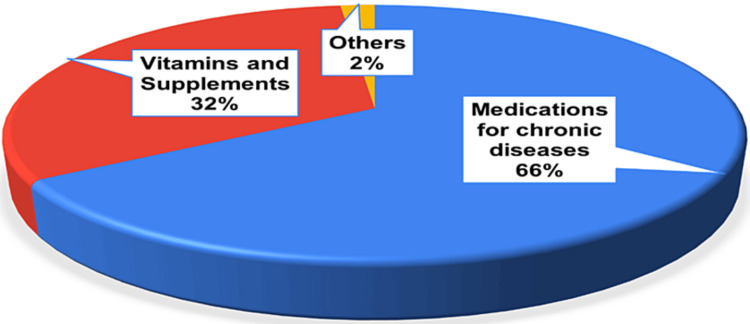
Medicines used by the participants Most participants used medications for chronic diseases, followed by supplements, while very few used other uncategorized medicines.

The survey revealed that the participants relied mainly on medications to manage chronic health issues. Figure 2 shows that a substantial majority (n = 443, 66.07%) reported using drugs for chronic conditions such as high blood pressure, high cholesterol levels, diabetes, asthma, and arthritis. Approximately n = 215, and 32.14% of the participants used vitamins and dietary supplements, indicating that around a third took supplements for potential health benefits. However, comparatively few (only n = 12, 1.79%) reported using other medicines not classified for chronic diseases or supplements.

Table 2 shows the self-reported symptoms evaluated to detect potential sleep disorders among the 670 participants. Difficulty falling or staying asleep showed that nearly half of the participants (n = 288, 43.0%) had difficulty sleeping, and n = 282, 42.1% woke up frequently at night. These symptoms suggested problems initiating or maintaining sleep, indicators of primary insomnia. Potential sleep-disordered breathing showed that a small proportion of the participants showed symptoms of sleep-disordered breathing. Only n = 88, 13.1% reported that they snored loudly; n = 80, 11.9% reported stopping breathing during sleep, and over 20% were unsure whether they snored. Disordered breathing symptoms affected n = 90, or 13.4%, of the participants.

**Table 2:** Questions for assessing sleep disorders among the participants Abbreviation: N: Number. (%): Percentage.

Questions		n	%
Do you find it difficult to sleep?	Yes	288	43.0
	No	382	57.0
Do you wake up frequently?	Yes	282	42.1
	No	388	57.9
Do you snore loudly?	Yes	88	13.1
	No	438	65.4
	I don’t know	144	21.5
Do you stop breathing during sleep?	Yes	80	11.9
	No	590	88.1
Do you wake up because of difficulty breathing?	Yes	90	13.4
	No	580	86.6
Do you wake up feeling completely paralyzed?	Yes	301	44.9
	No	369	55.1
Do you wake up in the morning and feel sleepy enough?	Yes	300	44.8
	No	370	55.2
Do you wake up with a dry throat?	Yes	300	44.8
	No	370	55.2
During the day, do you feel tired?	Yes	394	58.8
	No	276	41.2
Do you kick your leg while sleeping?	Yes	115	17.2
	No	555	82.8
Do you talk while sleeping?	Yes	117	17.5
	No	553	82.5

In restless leg syndrome and excessive daytime sleepiness (EDS) and their signs, almost half of the participants (n = 301, 44.9%) felt paralyzed, suggesting restless leg syndrome. Many also reported EDS, with n = 300, 44.8% still tired upon waking up, n = 300, 44.8% waking up with a dry throat, and n = 394, 58.8% feeling tired during the day.

Table 3 shows the assessment of sleepiness in various situations among the participants. Reading and watching TV showed that a significant proportion of the participants reported feeling sleepy while reading and watching TV. Specifically, n = 260, 38.8% reported a simple probability of feeling sleepy while reading, while n = 263, 39.3% reported the same probability while watching TV. Additionally, n = 103, 15.4% of the participants reported a high probability of feeling sleepy while reading, while n = 83, 12.4% reported the same probability while watching TV.

**Table 3:** Questions for assessing sleepiness among the participants Abbreviation: N: Number. (%): Percentage.

Do you feel sleepy in the following situations?		n	%
While reading	Zero probability	118	17.6
	High probability	103	15.4
	Simple probability	260	38.8
	Average probability	189	28.2
While watching TV	Zero probability	158	23.6
	High probability	83	12.4
	Simple probability	263	39.3
	Average probability	166	24.8
In a public place (e.g., meeting, party)	Zero probability	226	33.7
	High probability	85	12.7
	Simple probability	221	33.0
	Average probability	138	20.6
While driving for an hour without stopping	Zero probability	247	36.9
	High probability	98	14.6
	Simple probability	209	31.2
	Average probability	116	17.3
While lying down to rest in the afternoon	Zero probability	71	10.6
	High probability	239	35.7
	Simple probability	178	26.6
	Average probability	182	27.2
While sitting and talking to someone	Zero probability	417	62.2
	High probability	13	1.9
	Simple probability	193	28.8
	Average probability	47	7.0
While sitting quietly after lunch	Zero probability	104	15.5
	High probability	171	25.5
	Simple probability	203	30.3
	Average probability	192	28.7
While sitting in a car when it stops at a traffic light	Zero probability	446	66.6
	High probability	24	3.6
	Simple probability	149	22.2
	Average probability	51	7.6

In public places and driving, the participants were less likely to report feeling sleepy in public places, such as meetings, with only n = 85 and 12.7% reporting a high probability of sleepiness. However, n = 98, 14.6% of the participants reported a high probability of feeling sleepy when driving for an hour without stopping.

Rest and conversation

The participants reported feeling sleepy while lying down to rest in the afternoon, with n = 239 and 35.7% reporting a high probability of sleepiness. Most participants (n = 417, 62.2%) reported never feeling sleepy while sitting and talking to someone. Approximately n = 171, 25.5% of the participants reported a high probability of sleepiness while sitting quietly after lunch. When sitting in a car that stops at a traffic light (n = 24), 3.6% of the participants reported a high probability of sleepiness.

The findings suggest that situational factors are associated with sleepiness. Individuals may be more likely to feel sleepy during sedentary activities such as reading or watching TV. However, the level of sleepiness may vary depending on the situation. For instance, in this study, the participants were less likely to feel sleepy in public places but were more likely to feel sleepy while driving for an hour without stopping (Table 3).

**Figure 3:**
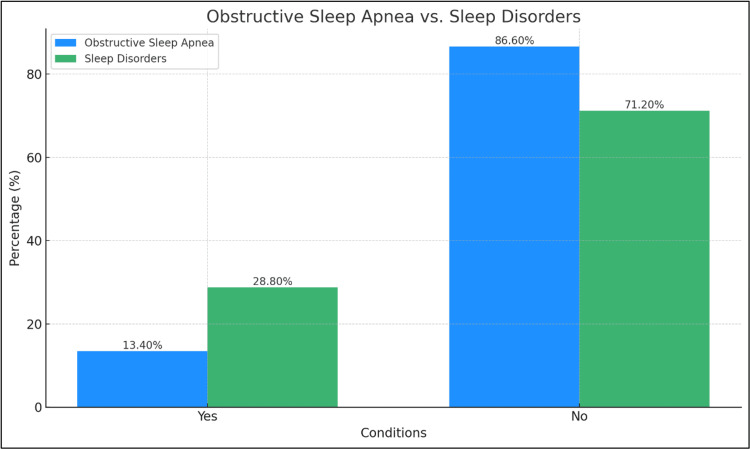
Prevalence of sleep disorders in general and obstructive sleep apnea

Figure 3 illustrates the prevalence of sleep disorders, focusing primarily on OSA and sleep disorders in general. It is observed that sleep disorders are relatively common, with a prevalence rate of 28.8% (n = 193). OSA, a specific type of sleep disorder, was found to have a 13.4% (n = 90) prevalence rate. On the flip side, most of the surveyed population did not report any sleep disorders. Specifically, n = 477, 71.2% of respondents reported no general sleep disorders, and a more significant proportion, n = 580, 86.6%, reported not having OSA.

Table 4 shows the association of sociodemographic and lifestyle factors and sleep patterns with the prevalence of sleep disorders. Sex did not appear to be significantly associated with sleep disorders (p = 0.877), as the proportions of the male and female participants without and with sleep disorders were similar (n = 181, 71.5% and n = 72, 28.5% for the male participants and n = 296, 71.0% and n = 121, 29.0% for the female participants, respectively). Conversely, age demonstrated a significant association with sleep disorders (p = 0.012). Among the different age groups, individuals aged ≥56 years had the highest prevalence of sleep disorders (n = 11, 45.8%). The prevalence gradually decreased in the younger age groups, reaching n = 83 and 24.1% for individuals aged 18-25. BMI was also significantly associated with sleep disorders (p = 0.043). The obese individuals had the highest prevalence of sleep disorders (n = 43, 38.4%). The prevalence decreased among the underweight individuals (n = 20, 21.5%). Occupation did not show a significant association with sleep disorders (p = 0.481). Regardless of occupation, the prevalence of sleep disorders remained relatively consistent across the participants: government sector employees (n = 36, 24.3%), private sector employees (n = 12, 33.3%), students (n = 75, 28.6%), and others or retired individuals (n = 70, 31.2%). Further, neck circumference demonstrated a significant association with sleep disorders (p = 0.034). Individuals with short or thin necks were likelier to experience sleep disorders, with n = 33 and 40.7% reporting such issues. Conversely, individuals with long or normal-sized necks had a lower prevalence of sleep disorders (n = 160, 27.2%).

**Table 4:** Association between sleep disorders and other factors Abbreviation: N: Number. (%): Percentage. *p-value is considered significant < 0.05. P-values were calculated using Pearson’s chi-square test.

Sociodemographic factors		Sleep disorders		p-value
		No n (%)	Yes n (%)	
Sex	Male	181 (71.5%)	72 (28.5%)	0.877
	Female	296 (71.0%)	121 (29.0%)	
Age, year	18–25	261 (75.9%)	83 (24.1%)	0.012*
	26–35	70 (61.4%)	44 (38.6%)	
	36–45	80 (69.0%)	36 (31.0%)	
	46–55	53 (73.6%)	19 (26.4%)	
	≥56	13 (54.2%)	11 (45.8%)	
Body mass index	Underweight	73 (78.5%)	20 (21.5%)	0.043*
	Normal weight	206 (73.3%)	75 (26.7%)	
	Overweight	129 (70.1%)	55 (29.9%)	
	Obesity	69 (61.6%)	43 (38.4%)	
Occupation	Government sector employee	112 (75.7%)	36 (24.3%)	0.481
	Private sector employee	24 (66.7%)	12 (33.3%)	
	Student	187 (71.4%)	75 (28.6%)	
	Others or retired	154 (68.8%)	70 (31.2%)	
Neck circumference	Long or normal	428 (72.8%)	160 (27.2%)	0.034*
	Short or thin	48 (59.3%)	33 (40.7%)	
	Middle size	1 (100.0%)	0 (0.0%)	
Do you smoke?	Yes	43 (56.6%)	33 (43.4%)	0.003*
	No	434 (73.1%)	160 (26.9%)	
Do you consume caffeine?	Yes	274 (65.9%)	142 (34.1%)	0.001*
	No	203 (79.9%)	51 (20.1%)	
Do you have a disease?	Yes	85 (58.2%)	61 (41.8%)	0.001*
	No	392 (74.8%)	132 (25.2%)	
Do you take medicines?	Yes	94 (63.1%)	55 (36.9%)	0.013*
	No	383 (73.5%)	138 (26.5%)	
Do you take naps during the day?	Yes	283 (74.3%)	98 (25.7%)	0.043*
	No	194 (67.1%)	95 (32.9%)	
Do you feel energized after a nap?	Yes	222 (76.8%)	67 (23.2%)	0.038*
	No	120 (65.2%)	64 (34.8%)	
Usual sleep hours per day	No specific time	36 (62.1%)	22 (37.9%)	0.071
	From 6 am to 12 pm	12 (63.2%)	7 (36.8%)	
	From 1 pm to 6 pm	4 (40.0%)	6 (60.0%)	
	From 7 pm to 12 am	276 (72.4%)	105 (27.6%)	
	From 1 am to 5 am	149 (73.8%)	53 (26.2%)	
Usual wake-up time per day	No specific time	16 (64.0%)	9 (36.0%)	0.839
	From 6 am to 12 pm	253 (71.9%)	99 (28.1%)	
	From 1 pm to 6 pm	37 (72.5%)	14 (27.5%)	
	From 7 pm to 12 am	4 (57.1%)	3 (42.9%)	
	From 1 am to 5 am	167 (71.1%)	68 (28.9%)	
How often do you get up to go to the bathroom?	Once at most	334 (77.0%)	100 (23.0%)	0.001*
	Twice at most	81 (60.4%)	53 (39.6%)	
	More than twice	16 (36.4%)	28 (63.6%)	
	Not once	45 (81.8%)	10 (18.2%)	

Several lifestyle factors were also found to be associated with sleep disorders. Smoking (p = 0.003), consuming caffeine (p = 0.001), having a disease (p = 0.001), taking medicines (p = 0.013), taking naps during the day (p = 0.043), and feeling energized after a nap (p = 0.038) all displayed significant associations with sleep disorders. The participants who smoked, consumed caffeine, had a disease, and took medicines exhibited a higher prevalence of sleep disorders than their counterparts. In contrast, those who did not take naps during the day and felt energized after a rest showed a higher prevalence of sleep disorders than their counterparts.

Moreover, the relationship between sleep patterns and sleep disorders. The usual daily sleep hours did not significantly correlate with sleep disorders (p = 0.071). Similarly, the usual wake-up time per day was not significantly associated with sleep disorders (p = 0.839). However, the frequency of getting up to go to the bathroom was significantly associated with sleep disorders (p = 0.001). The participants who reported getting up more frequently at night had a higher prevalence of sleep disorders than the others.

**Figure 4:**
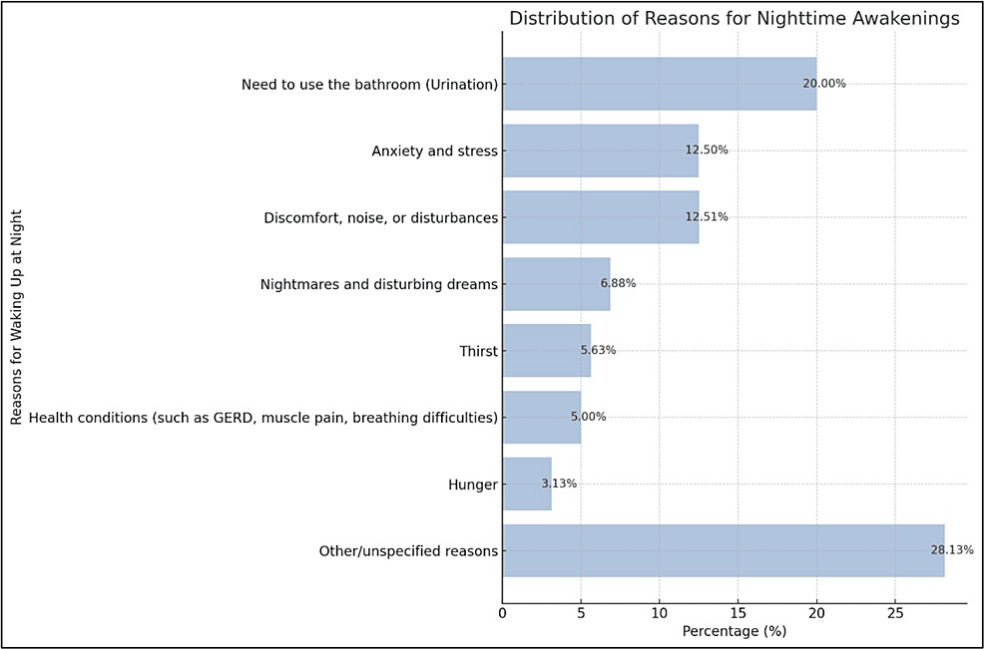
Reasons for waking up from sleep at night

Figure 4 illustrates the main reasons the participants woke up from sleep at night. The need to use the bathroom (urination) was the most common reason for waking up at night, with n = 134 and 20% of the respondents reporting this as the reason. Conversely, approximately n = 84, 12.5% of the participants reported anxiety and stress as the reasons. Other frequent explanations for waking up at night included discomfort/noise/disturbances (n = 84, 12.51%), nightmares/disturbing dreams (n = 46, 6.88%), and health conditions such as GERD, muscle pain, and breathing difficulties (n = 33.5, 5%). Thirst and hunger accounted for n = 38, 5.63%, and n = 21, 3.13% of the responses, respectively. A sizable proportion (n = 188, 28.13%) selected “other/unspecified reasons” for waking up from sleep at night.

## Discussion

This study provides insightful data about the prevalence and associated risk factors of sleep disorders within the Jazan population. Most participants were women, were young to middle-aged adults, and had normal or long neck sizes. Approximately 28.8% of the respondents had a sleep disorder, with 13.4% experiencing OSA. These results align with previous reports from Saudi Arabia: A high prevalence of sleep disorders was reported in Makkah among medical students (73.8%) and patients with type 2 diabetes (63.7%) [19,20]. In Taif, 15.2% had a high risk of developing OSA [21]. A recent study also showed that 24.0% of women and 47.3% of men were diagnosed with OSA [22].

In the present study, primary insomnia and EDS were significant symptoms reported by many respondents, suggesting potential sleep disorders. This finding is consistent with previous data indicating a 10%-30% prevalence of insomnia in the general population [23,24]. Likewise, over 40% of our participants reported tiredness upon waking up or during the day, corroborating past findings of an EDS prevalence of around 20% [25]. Furthermore, we observed that 13% of the participants showed signs of sleep-disordered breathing, while nearly 45% reported symptoms of restless leg syndrome. In a Riyadh study, 70.3% of individuals had trouble going to bed, and 58.1% had sleep-onset delay [26]. In an Al-Ahsa study, 97.5% of participants were familiar with sleep paralysis, a common sleep problem [27]. Frequent nightmares (39.1%), insomnia (41.3%), and suspected sleep-disordered breathing (79.3%) were common in at-risk teens [28]. In a recent study, 15.8% of women and 9.3% of men had insomnia, while 3.7% of women and 2.2% of men had restless leg syndrome [22].

Our analysis also highlighted the influence of sedentary activities such as reading and watching TV on sleepiness possibly owing to decreased stimulation. In contrast, being in public places and driving were associated with a lower prevalence of sleepiness, suggesting the heightened alertness required for these activities [29]. Leisure-time physical activity and other sedentary behavior were linked to EDS in middle-aged and older persons [30]. Nonetheless, the high prevalence of sleepiness while driving (14.6%) raises safety concerns, considering the increased risk of accidents associated with drowsy driving [31]. In a previous study, sleepiness while driving was prevalent among 31.5% of truck drivers [32].

We also identified obesity, older age, short neck, smoking, caffeine intake, health conditions, and medication use as factors associated with a higher prevalence of sleep disorders. These factors align with known risk factors, such as obesity [33-35], aging [36], anatomical issues [37], and certain lifestyle factors [34,38]. In Riyadh, it was found that insufficient sleep was commonly associated with obesity (39.1%), hypertension (33.9%), depression (4.3%), and asthma (17.3%) [39].

Frequent nocturnal urination interruptions were also found to be associated with sleep disorders in this study; these interruptions were likely attributable to underlying health conditions. These results are consistent with previous epidemiological reports [40,41]. The present data underscore the importance of lifestyle changes and adequate treatment of medical conditions to improve sleep health. The high prevalence of waking up to use the bathroom highlights the prevalence of nocturia, which increases with age and the presence of medical conditions [42]. It also highlights the detrimental effects of anxiety and stress on sleep [43]. Sleep interruptions can lead to fragmented sleep and worse outcomes [44].

In general, this study provides valuable insights into the burden of sleep disturbances and disorders in the Jazan population. The results emphasize the need for increased clinical and public health initiatives to enhance sleep health through modification of risk factors and screening, diagnosis, and evidence-based treatment of sleep disorders. Raising awareness, encouraging consultations with sleep specialists, promoting healthy sleep habits, improving identification and diagnostics, and utilizing a multidisciplinary treatment approach could help reduce the impact of sleep disorders prevalent among the study participants.

Limitations

This study has some limitations. First, the study relied solely on self-reported data, making it susceptible to recall and reporting biases. More objective sleep measures could yield more accurate findings. Second, the sample was imbalanced, with a higher proportion of women and students, making it less representative of the general population. Future studies should aim for more sex-balanced and diverse samples. Third, the study could only associate lifestyle and demographic factors with sleep disorders and could not establish causation. Experimental and longitudinal study designs are required to determine causality. Fourth, as a cross-sectional study, it captured data at a single time point and could not track changes over time. Longitudinal studies would provide greater insights. Finally, the study’s regional focus limits the generalizability of the results to other populations and cultures. Future research should include more diverse international samples.

## Conclusions

This study revealed a high prevalence of sleep disorders, affecting over a quarter of respondents in the surveyed population. The data underscores the need for greater awareness and education about sleep health. Targeted strategies focused on moderating lifestyle factors like obesity, smoking, and caffeine use could help address the high rates of insomnia symptoms and sleep disturbances reported. Situational factors like sedentary activities and driving also contributed to increased daytime sleepiness among participants.

Our findings highlight that multiple demographic, medical, and lifestyle factors impact sleep quality. Promoting sleep hygiene through managing related health conditions and improving public knowledge of the fundamentals of healthy sleep is important for well-being. Comprehensive interventions to ameliorate the high burden of sleep disorders are the need of the hour, as good sleep is essential for mental and physical health. This study sheds light on the prevalence of sleep problems and their multifactorial nature, underscoring the importance of holistic strategies addressing various contributing factors to improve sleep outcomes and quality of life.
